# Evaluating AI-Based Mitosis Detection for Breast Carcinoma in Digital Pathology: A Clinical Study on Routine Practice Integration

**DOI:** 10.3390/diagnostics15091127

**Published:** 2025-04-28

**Authors:** Clara Simmat, Loris Guichard, Stéphane Sockeel, Nicolas Pozin, Rémy Peyret, Magali Lacroix-Triki, Catherine Miquel, Arnaud Gauthier, Marie Sockeel, Sophie Prévot

**Affiliations:** 1 Primaa, 75002 Paris, France; 2 Hôpital Bicêtre (AP-HP), Paris-Saclay University, 94270 Kremin-Bicêtre, France; 3 Gustave-Roussy Cancer Campus—Grand Paris, 94800 Villejuif, France; 4 Hôpital Saint-Louis (AP-HP), Paris Cité University, 75010 Paris, France; 5 Institut Curie, PSL University, 75005 Paris, France

**Keywords:** invasive breast carcinoma, mitoses, hotspots, digital pathology, WSI, artificial intelligence, deep learning mitotic score reproducibility, clinical study

## Abstract

**Background/Objectives:** An accurate assessment of mitotic activity is crucial in the histopathological diagnosis of invasive breast carcinoma. However, this task is time-consuming and labor-intensive, and suffers from high variability between pathologists. **Methods**: To assist pathologists in routine diagnostics, we developed an artificial intelligence (AI)-based tool that uses whole slide images (WSIs) to detect mitoses, identify mitotic hotspots, and assign mitotic scores according to the Elston and Ellis grading system. To our knowledge, this study is the first to evaluate such a tool fully integrated into the pathologist’s routine workflow. **Results:** A clinical study evaluating the tool’s performance on routine data clearly demonstrated the value of this approach. With AI assistance, pathologists achieved a greater accuracy and reproducibility in mitotic scoring, mainly because the tool automatically and consistently identified hotspots. Inter-observer reproducibility improved significantly: Cohen’s kappa coefficients increased from 0.378 and 0.457 (low agreement) without AI to 0.629 and 0.726 (moderate agreement) with AI. **Conclusions:** This preliminary clinical study demonstrates, for the first time in a routine diagnostic setting, that AI can reliably identify mitotic hotspots and enhance pathologists’ performance in scoring mitotic activity on breast cancer WSIs.

## 1. Introduction

Integrating slide digitization and AI-based solutions into routine pathology holds great promise for enabling faster, more accurate, and more reproducible diagnoses, ultimately improving patient care. In breast pathology, numerous studies have successfully applied computer vision tools to analyze whole slide images (WSI) of breast carcinoma specimens. Some research studies focus on lesion detection [1,2,3] while others aim to identify specific biomarkers such as tumor-infiltrating lymphocytes [4,5]. Diagnosing lesions comprehensively often involves multiple complex and time-consuming tasks.

For example, in invasive carcinoma of no specific type (IC-NST), pathologists must identify the area with the highest density of dividing cells (mitoses)—known as a mitotic hotspot (MH)—and count the mitoses within this region. The mitotic score (MS), an integral component of the Elston and Ellis histoprognostic grading system [6], is derived from the mitotic count (MC) performed in these hotspots. The MS is categorized into three grades (1, 2, and 3). However, mitotic counting is a tedious and time-consuming process. A study by Rakha et al. [7] highlighted significant inter-observer variability in MC, noting that disagreements often arise from differences in hotspot selection. Because mitoses are small structures requiring high magnification (40×) during examination, thoroughly inspecting the entire tumor area is challenging. Furthermore, identifying mitotic figures is subjective, as they can be easily confused with degenerating cells, apoptotic bodies, or necrotic debris. The introduction of AI tools in this field offers the potential to enhance reproducibility, speed, and ease of assessment.

Automatic mitosis detection using AI-based tools could reduce variability, improve accuracy, and save time in pathology workflow. A recent study by Berbís et al. [8] predicted that AI-based MC would likely be standard practice by 2030. In 2023, the French Society of Pathology (SFP) surveyed 165 French pathologists regarding the potential contributions of AI to the field: 56.4% identified mitotic activity assessment as the most challenging aspect of the Elston and Ellis scoring system, while 84.2% indicated a need for diagnostic support solutions, including automated quantitative measurements like mitotic counting.

Numerous studies have focused on automatic mitosis detection, with many applying standard computer vision techniques to detect or classify mitoses on WSIs. For instance, in the works of Irshad [9], F. Boray Tek [10] employed conventional image processing techniques such as active contour models and thresholding to locate nuclei and handcrafted features to determine mitotic status. As Mathew et al. [11] stated, deep learning techniques gained prominence in the mid-2010s. Most studies employ deep networks for mitotic figure localization [12,13,14] or segmentation [15,16,17,18], followed by a classification model to remove false positives. Typically, these algorithms are calibrated and tested on public datasets such as CCMCT [19], MITOS [20], or MITOS-ATYPIA [21]. The MIDOG2021 [22] challenge also released a public dataset with mitoses labeled from WSI originating from various scanners.

Recent studies on mitosis detection in WSIs have shown significant advancements in accuracy and efficiency through deep learning models. Notable approaches include a combination of Faster R-CNN and YOLOv5, which achieves an F1-score of 84% using a large annotated dataset and providing an end-to-end web-based platform for image analysis and diagnosis [23,24]. Another innovative method, “Mitosis Detection, Fast and Slow”, employs a two-stage framework for candidate segmentation and refinement, enhancing sensitivity and computational efficiency by initially processing downscaled images and refining them with deeper Convolutional Neural Networks (CNNs) [25]. Recently, Wang et al. [26] proposed a novel two-stage deep learning method combining attention-enhanced feature fusion and optimized residual blocks, achieving state-of-the-art performance on both the ICPR 2012. Additionally, efforts to address domain shifts in WSIs through color augmentation and stain normalization have been explored to improve model generalizability across different imaging conditions [22].

However, despite improved performance on benchmark datasets, the clinical applicability and impact of these tools in real-world diagnostic settings have not been thoroughly investigated. Most existing studies focus on analytical validation using standard metrics (precision, recall, F1-score), which may not adequately reflect performance in actual clinical practice. Given the high inter-observer variability in mitotic counting, these metrics can be unstable. They are sensitive to inconsistencies (‘noise’) in the ground truth annotations, where the identification of mitoses itself is subjective. Therefore, these metrics may not reliably indicate the tool’s true clinical utility and could reflect overfitting to annotation biases rather than generalizable performance. The following section reviews existing clinical studies assessing AI-assisted mitotic scoring in diagnostic practice. Then, the next sections will focus on the contribution of this study.

## 2. Related Works

Several clinical studies have attempted to assess the practical benefits of integrating AI-based mitosis detection into routine pathology workflows. Pantanowitz et al. [27] showed that pathologists were more accurate and efficient in quantifying mitotic figures in digital images of IC-NST with AI assistance. However, that study performed counts on pre-selected fields of IC-NST and did not assess the AI’s ability to help find relevant mitotic hotspots—a crucial step in determining the mitotic score. A study by Veta and Van Diest [28] compared mitotic counting results with and without AI assistance by two pathologists using both WSI and light microscopy. It showed that mitotic counts on WSIs with AI assistance could be non-inferior to counts performed using traditional light microscopy. However, pathologists had to manually delineate the area of interest (ROI) as it was not automatically proposed. Balkenhol’s study [29] demonstrated a strong agreement between two pathologists in determining the mitotic score using AI-identified mitotic hotspots. However, the assessment was limited to a predefined 2 mm^2^ hotspot area rather than the entire slide, which does not reflect typical clinical practice. These prior studies have three main limitations. First, mitotic detection is restricted to limited regions of IC-NST, requiring manual selection of ROI. For AI assistance to be truly effective, mitosis detection should be integrated into a comprehensive WSI processing workflow that locates IC-NST, detects mitoses, proposes hotspots, and facilitates counting within these regions. Second, public datasets are often composed of research-quality slides with clear mitotic figures, whereas routine data may be more challenging for mitotic detection. Third, the extent to which these tools actually improve pathologists’ performance in practice has not been adequately evaluated.

In this context, our work seeks to address these limitations via the following:

- Providing evidence that AI assistance can reduce inter-observer variability in real-world clinical settings;
- Integrating a comprehensive deep learning pipeline—for IC-NST localization, mitosis detection, and hotspot identification on whole-slide images—into a user-friendly interface seamlessly embedded within the pathologist’s workflow, with no need for manual region-of-interest (ROI) selection or intervention;
- Conducting a clinical evaluation comparing pathologists’ performance with and without AI assistance;
- Performing a subgroup analysis to identify specific scenarios where AI assistance offers the most significant benefit.

## 3. Materials and Methods

### 3.1. Data Description

The data used to train and test the models originate from two complementary sources of WSI: the MIDOG 2021 challenge dataset [22] and a local dataset from Bicêtre Hospital. Using these two sources aimed to increase the variability in mitotic patterns, tissue appearance, and slide quality, thereby enhancing the pipeline’s robustness and generalizability.

The MIDOG 2021 dataset consists of breast cancer WSIs stained with routine hematoxylin-eosin (HE) and scanned using three different scanners—Hamamatsu XR Nano-Zoomer 2.0, Hamamatsu S360, and Aperio ScanScope CS2. The training set includes 1721 annotated mitotic figures and 2714 hard negatives (non-mitotic figures with high visual similarity). For this study, only the annotated mitotic figures were used to train the detection model. As with many challenge datasets, MIDOG slides were curated to maintain high quality, with thin tissue sections and minimal artifacts. While valuable for algorithm development, these curated datasets may not fully represent the heterogeneity and imperfections common in routine clinical practice.

To address this gap and ensure the pipeline’s applicability in real-world conditions, an additional dataset was built in collaboration with Bicêtre Hospital. This dataset consists of 32 routine hematoxylin-eosin-safran (HES)-stained WSIs scanned at ×20 magnification with 3DHistech P250 and P1000 scanners (digital zoom 1.6×). These WSIs represent daily diagnostic practice, capturing the variability in staining intensity, section thickness, and presence of artifacts typical of clinical workflows.

To minimize annotation errors (‘noise’)—especially considering the known difficulty in consistently identifying mitoses on digital versus glass slides [30,31,32,33,34]—we applied a two-step expert annotation protocol. First, a senior pathologist exhaustively labeled mitosis-like figures on each slide, including typical mitoses, atypical forms, and potential imposters. Then, a consensus review was conducted with a second experienced pathologist, incorporating both digital and glass slide evaluations to refine the annotations. This process aimed to improve annotation consistency and reduce false positives, particularly for ambiguous figures. Although this approach improved dataset quality, some annotation errors likely remain due to the inherent challenges of mitosis identification on WSIs. Annotations were carried out using the Cytomine platform [35], resulting in a curated dataset of 1677 mitotic figures.

This rigorous protocol was specifically designed to reduce inter-observer variability and increase annotation reliability, ensuring that the training data reflects expert-level consensus under realistic clinical conditions.

### 3.2. Pipeline Description

**Figure 1:**
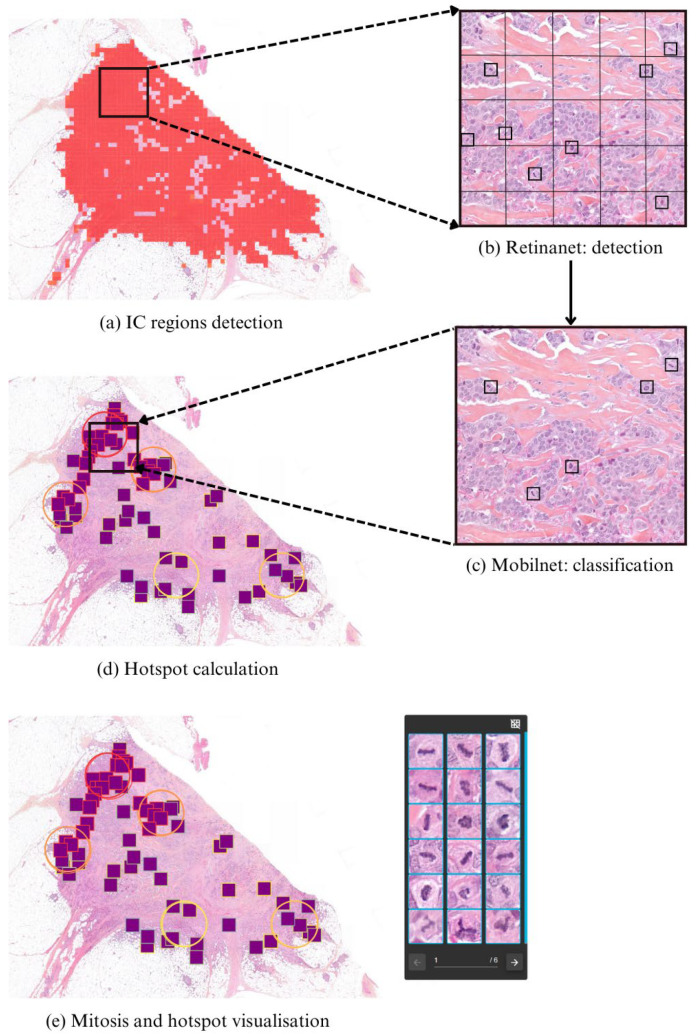
Overview of the detection pipeline. First, IC regions are identified (**a**) and divided into 256 × 256 pixel patches at 20× magnification. Within these IC patches, mitosis localization follows a two-stage process: a RetinaNet detector (**b**) first identifies mitosis-like objects, which are then passed to a classifier (**c**) for validation. Only objects exceeding a confidence threshold are retained and displayed as purple boxes. Finally, the regions with the highest mitotic density (hotspots) are automatically identified and displayed as circles (**d**). The results can be visualized using the in-house software Cleo (**e**).

The proposed detection pipeline is a multi-stage process designed to assist pathologists in mitotic scoring by automatically identifying mitotic figures and the most mitosis-rich regions (MH) within IC-NST areas (see Figure 1).

#### 3.2.1. IC-NST Region Detection

In the first step of the pipeline, regions corresponding to invasive carcinoma of no special type (IC-NST) are automatically detected using a patch-based classification model. This model uses an EfficientNetB1. It operates on patches of 256 × 256 pixels extracted at 5× magnification from whole-slide images, enabling efficient identification of tumor regions at a low resolution. Once IC-NST areas are detected, they define the spatial scope for all subsequent mitotic analyses. These identified tumor regions are then further tiled into non-overlapping patches of 256 × 256 pixels at 20× magnification, which are passed to the mitosis detection module in the next stage.

#### 3.2.2. Mitosis Candidate Localization

Each patch identified as containing IC-NST is processed through a two-stage mitosis localization pipeline designed to ensure both high sensitivity and precision.

- First, a RetinaNet-based object detector [36] analyzes 50 × 50 pixel crops to localize mitosis-like candidates.
- These candidate regions are then evaluated by a Mo-bileNetV2-based classifier [37], which determines whether each candidate corresponds to a true mitotic figure.
- Only predictions that exceed a predefined confidence threshold are retained.

This cascaded approach leverages RetinaNet’s strength in object localization and Mo-bileNetV2’s efficiency in filtering out false positives, ensuring robust and accurate mitosis detection.

#### 3.2.3. Hotspot Computation

To identify regions of diagnostic interest, we compute mitotic hotspots (MHs)—circular areas with the highest estimated mitotic activity. Each patch, *p*, containing a mitotic figure is assigned a hotspot score $h_{p}$, defined as follows:

$$h_{p}=M_{core}\left(p\right)+\epsilon \times M_{context}(p)$$

where

- $h_{p}$ is the hotspot score assigned to patch *p*.
- $M_{core}(p)$ is the number of mitoses within a core circular region 𝒞_core (e.g., 1 mm^2^) centered on patch *p*.
- $M_{context}(p)$ is the number of mitoses within a broader circular context 𝒞_context (e.g., 2 mm^2^), excluding the core region.
- ε ∈ [0, 1] is a tunable weight controlling the influence of the surrounding mitotic activity.

This scoring strategy reflects expert pathologists’ preferences for biologically relevant mitotic patterns and is summarized in pseudocode below.

**Input:**
  - *M* = {(x_1_, y_1_), …, (x_n_, y_n_)}  // mitoses
  - *P* = {(x_1_, y_1_), …, (x_k_, y_k_)}  // patch centers
  - *r*_1_ = radius (1 mm^2^), *r*_2_ = radius (2 mm^2^)
  - ε = surrounding weight
**Function:**
Count(*c*, *r*, *M*):
  return |{ m ∈ *M*: dist(m, *c*) ≤ *r* }|
**Main:**
H ← {}
for p ∈ P:
  n_1_ ← Count(*p*, r_1_, M)
  n_2_ ← Count(*p*, r_2_, M)
  H[*p*] ← n_1_ + ε·(n_2_ − n_1_)
return sort_desc(H)

#### 3.2.4. Visualization and Clinical Support

Detected mitotic figures and the top-ranking MHs are displayed in the in-house viewer Cleo, where the 2 mm^2^ regions are highlighted for pathologist review and scoring (Appendix C).

### 3.3. Data and Training

#### 3.3.1. Datasets

In the following, the WSIs used for training and testing are referred to as train slides and test slides, respectively. The corresponding patch datasets used to train and evaluate the detection networks are termed the *detection train set* and *detection test set*. A similar naming convention is applied to the classification datasets.

As summarized in Table 1, the *train slides* include 150 slides from MIDOG and a subset of 12 Bicêtre slides, while the *test slides* consist exclusively from Bicêtre data.

For the detection task, the train and test slides were segmented into 256 × 256 pixel patches at ×20 magnification, with the following:

- For the training set: 2791 patches containing at least one mitosis.
- For the testing set: 1341 patches without mitosis, and 146 patches with mitosis and 24,716 patches without mitosis.

For the classification task, false positives detected by the RetinaNet detection algorithm in the *detection train set* were labeled as negative class instances. The same approach was applied to the *detection test set* to generate artifacts for the classification test set. However, due to the inherent challenges of manual annotation, some mitotic patterns may have been overlooked by the annotators. As a result, the artifact set contains true mitotic patterns that were detected by RetinaNet but were not originally annotated, leading to false positives. To refine this artifact dataset, only objects with a detection confidence below a predefined threshold are retained, ensuring the integrity of the classification datasets. This filtering step has been shown to enhance the performance of the classification network. After processing, the final classification datasets consisted of the following:

- Training set: 3106 mitoses and 8638 artifacts.
- Testing set: 153 mitoses and 5081 artifacts.

All classification patches were extracted with a size of 50 × 50 pixels at 20× magnification.

#### 3.3.2. Data Augmentation

Data augmentation techniques, including color jitter, cutout, blur, and geometric transformations, were randomly applied for both detection and classification. They significantly improved model generalization and robustness to variations in staining, artifacts, and imaging conditions

#### 3.3.3. Training Configuration

The RetinaNet model was trained with a standard L1-loss for bounding box regression and a focal loss [36] for instance classification. Optimization was performed using stochastic gradient descent with a piecewise constant decay learning rate. The classifier, based on the MobileNetV2 [37] architecture, is trained with a binary cross-entropy loss and the Adam [38] optimizer. Both models have been optimized to achieve the best possible performance on a validation set, with loss functions guiding parameter selection for optimal results, as described in Table 2.

#### 3.3.4. Analytical Validation of the Detection Pipeline

We previously assessed the analytical performance of this detection pipeline in an earlier study by Guichard et al. [39]. That work focused on evaluating the algorithm’s ability to detect mitotic figures using standard metrics such as precision, recall, and F1-score. This prior work constitutes the analytical validation of the model, based on expert-annotated slides containing 153 mitotic figures across 24,862 IC-NST patches. The two-stage detector and a MobileNetV2-based classifier achieved a recall of 56.2% and precision of 12.6% at the detection stage and recall of 43.8% and precision of 27.6% after classification refinement.

To evaluate the generalizability of the model, the pipeline was also tested on two public datasets widely used in the field: MIDOG 2022 [40] and MITOS-ATYPIA [21]. The detection metrics across all datasets are summarized in Table 3. Performance on these external datasets confirmed the model’s robustness across varying staining conditions, scanner types, and annotation protocols.

However, interpreting these common metrics for mitosis detection is challenging due to the high inter-observer variability in the reference annotations. Mitotic figures are inherently difficult to identify consistently, even among expert pathologists, making the ground truth itself noisy. As a result, such metrics may reflect overfitting to annotation artifacts rather than generalizable diagnostic performance.

To provide a broader view of the algorithm’s robustness, Guichard et al.’s study [39] also reported an Intra-class Correlation Coefficient (ICC) of 0.644 between the algorithm and expert mitotic scoring, which is in the same range as the ICC of 0.716 (95% CI: [0.531–0.833]) observed between pathologists without AI assistance in the present study. This suggests that the algorithm’s agreement with human experts is comparable to inter-expert reproducibility. A similar evaluation strategy was employed in Pantanowitz et al.’s study [27], which also assessed algorithm–expert agreement using ICC, further validating the relevance of this approach for benchmarking AI assistance in mitotic scoring.

Further details on the methodology and comprehensive results of the analytical validation study are provided in Appendix A.

### 3.4. Design of the Clinical Study

#### 3.4.1. Patients and Tissue Selection

The study was conducted on 50 specimens randomly selected between February 2020 and June 2021 from the pathology department of Bicêtre Hospital, not used during training or testing, and included a balanced combination of biopsies and surgical specimens. Table 4 summarizes the patient and tumor characteristics.

Biopsies underwent fixation in 4% neutral buffered formalin for a minimum of 6 h, while surgical specimens were sampled following fixation in 4% buffered formalin for 24 to 48 h. Subsequently, the specimens underwent dehydration and impregnation using a Sakura Tissue-Tek^®^ VIP^®^ machine in accordance with departmental protocol. Paraffin embedding procedures differed for surgical specimens and biopsies, with automated embedding performed on a Sakura Tissue-Tek^®^ AutoTEC^®^ and manual embedding on a Sakura Tissue-Tek^®^ system, respectively. Sections, cut to a thickness of 3 µm using a Leica or Microm microtome, were mounted onto SuperFrost™ glass slides and dried at 60 °C for 30 min before undergoing staining with Hematoxylin-Eosin-Safran on a Leica ST5020^®^ automated system. Slides were then mounted using Pertex^®^ mounting medium on a Leica CV5030^®^ and dried for a minimum of 5 min in the machine.

Case scanning was conducted using two 3DHISTECH slide scanners, P1000 for surgical specimens and P250 for biopsies, each equipped with two Plan-Apochromat lenses (×20 and ×40) and an Adimec QUARTZ Q-12A180 camera, providing a resolution of 4096 × 3072 pixels (pixel size: 5.5 μm × 5.5 μm) for digital magnification by 1.6.

All 50 cases were scanned in .mrxs format at a resolution of 0.24 μm/pixel using the ×20 lens with a digital magnification factor of 1.6, following a protocol consistent with routine diagnostic practices within the department.

#### 3.4.2. Study Design

First, the study involved three expert pathologists with substantial experience in digital pathology, hereafter referred to as expert annotators. Each expert independently performed MC on all 50 WSIs, strictly following the assessment protocol described by Ibra-him et al. [30]. This included locating the region with the highest mitotic activity—commonly known as the mitotic hotspot—and manually counting mitotic figures at high magnification. In 17 of the 50 slides, the experts disagreed on the MS. These discrepancies were resolved through a collaborative consensus meeting, during which all experts jointly reviewed the slides and agreed on a final mitotic score for each case. These consensus scores served as a reference standard for the study.

Next, two junior pathologists—referred to as investigators—were tasked with evaluating the same set of slides. Each investigator independently performed both MC and MS for all slides, using the Elston and Ellis grading criteria. To assess the impact of AI assistance, a crossover study design was implemented:

- In the first session, each investigator reviewed half of the slides without access to the AI tool and the remaining half with AI assistance.
- Then, after a washout period of several weeks (to minimize recall bias), the slide sets were switched: each investigator re-evaluated the cases, now using the opposite condition (i.e., slides previously reviewed with AI were now reviewed without, and vice versa).

When using the AI tool, investigators were shown the predicted mitotic hotspot and associated mitotic figures. However, they retained full autonomy to select the region they deemed most representative of mitotic activity; they were not required to follow the AI suggestions. This design ensured that AI served as a decision support tool rather than dictating the outcomes.

Importantly, both investigators were blinded to each other’s assessments and the expert consensus scores.

**Figure 2:**
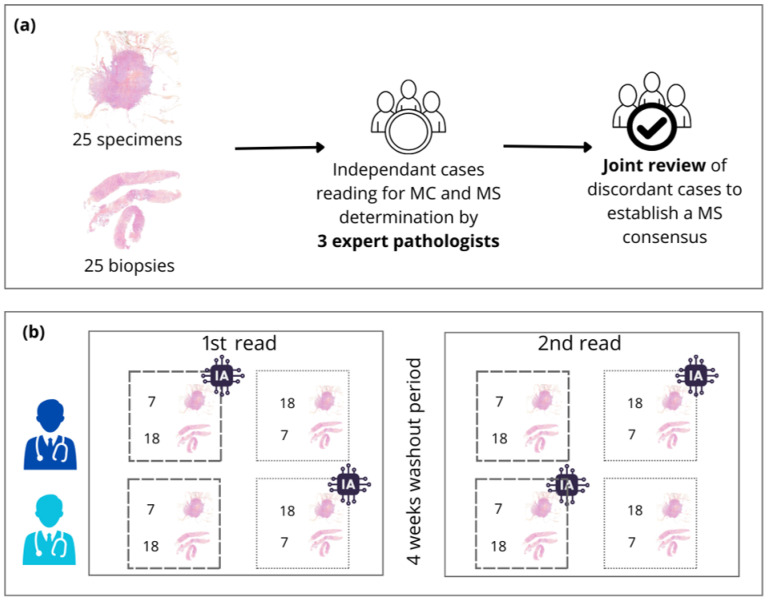
(**a**) Process for consensus establishment. (**b**) Process for pathologist readings with and without AI assistance.

This rigorous protocol ensured unbiased, independent assessments under both experimental conditions. The complete study workflow is illustrated in Figure 2.

#### 3.4.3. Statistical Analysis

The analysis was conducted in two main stages. The first objective was to evaluate the accuracy of MS. Accuracy was defined as the proportion of WSIs where the mitotic score assigned by the investigator matched the expert consensus (ground truth). This metric was calculated under two conditions: (i) when investigators had access to AI assistance and (ii) when they performed the evaluation without AI assistance.

The second objective focused on assessing inter-observer agreement between the two investigators, both for MS and MC. For MS, agreement was quantified using Cohen’s kappa coefficient, which provides a measure of consistency beyond chance. In addition to kappa values, 95% confidence intervals (CIs) were reported. To complement this, the ICC was also computed to capture the overall reliability of scoring between investigators.

For MC, agreement was assessed by evaluating whether both investigators selected overlapping mitotic counting regions on the same slide. The proportion of slides where their selected regions intersected served as a metric for spatial agreement in hotspot selection.

A central goal of the study was to investigate whether discrepancies in mitotic counts could be attributed primarily to differences in region selection between pathologists and whether the proposed AI tool could help mitigate this source of variability.

For interpretation of reproducibility metrics, Koo and Li’s 2016 guidelines [41] were used to interpret ICC values related to MC, while McHugh’s 2012 scale [42] was applied for interpreting Cohen’s kappa values related to MS reproducibility. All statistical analyses, including the computation of accuracy, kappa coefficients, ICCs, and confidence intervals, were performed using R software (version 4.0.4) and RStudio (version 2022.02.2 Build 485).

## 4. Results

### Study Outcomes

**Figure 3:**
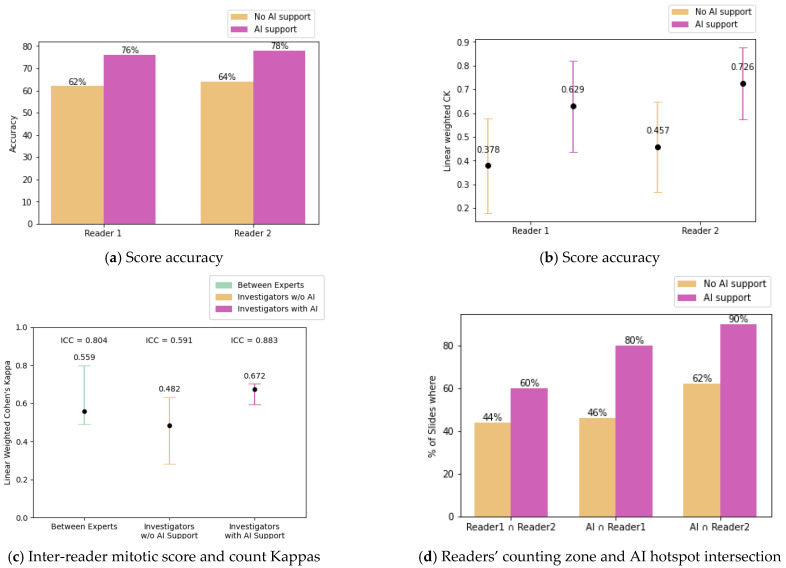
(**a**) Mitotic score accuracy for each reader compared to the ground truth consensus, with and without AI assistance. (**b**) Linear weighted Cohen’s Kappa values for each reader compared to consensus. (**c**) ICC and linear weighted Cohen’s Kappa values computed between readers with and without AI assistance and between experts. (**d**) Percentage of slides where both readers’ counting zones intersect (Reader1 ∩ Reader1), with and without AI, and the percentage of slides where an AI hotspot intersects a reader’s counting zone, for both readers, with and without AI assistance (AI ∩ Reader1 and AI ∩ Reader2).

This clinical study, conducted using a crossover scheme with and without AI assistance on WSIs of breast IC without predefined ROIs, demonstrated an improvement in MS accuracy. With AI assistance, MC accuracy improved significantly: accuracy increased from 62% to 76% for investigator 1 and from 64% to 78% for investigator 2 when compared against the expert consensus. (Figure 3). Reproducibility also improved, as shown by the linear weighted Cohen’s kappa values, which increased from 0.378 to 0.629 for investigator 1 and from 0.457 to 0.726 for investigator 2 (Figure 3a,b; raw confusion matrices can be found in Appendix B).

Reproducibility of the MS between investigators also improved with AI assistance, as measured by the weighted Cohen’s kappa (CK), which increased from 0.482 to 0.672. This increase is further supported by the ICC evolution from 0.591 (CI: [0.375–0.746]) to 0.883 (CI: [0.803–0.932]) and their confidence intervals. (Figure 3c, raw confusion matrices can be found in Appendix B).

Agreement on the location of counting zones (defined by intersecting areas chosen by each investigator) increased from 44% to 60% with AI assistance (Figure 3d). In 18% of cases, scores changed with AI use (8 and 10 for investigators 1 and 2, respectively).

Metrics were further analyzed for subgroups of slides with the same ground truth MS (1, 2, or 3). Given the differences in tumor size between biopsies and surgical specimens, results were also examined separately for each specimen type, as detailed in Table 5.

AI assistance led to an improvement in accuracy and kappa coefficient for WSI with an MS of 2 from 5.56% to 33.3% and from 0 to 0.31, respectively. For WSI with an MS of 3, accuracy improved from 29.17% to 66.67%, and kappa increased from 0.31 to 0.47. No change in accuracy or kappa was observed for WSIs with a mitotic score of 1.

Improvements were also observed across specimen types. For biopsies, accuracy increased from 60% to 72%, with kappa rising from 0.17 to 0.53. For surgical specimens, accuracy improved from 66% to 82%, and kappa from 0.55 to 0.73.

Among all subgroups, the most significant improvement in the intersection of counting zones between investigators was observed for WSIs with a mitotic score of 3, increasing from 33.3% to 66.7%.

**Figure 4:**
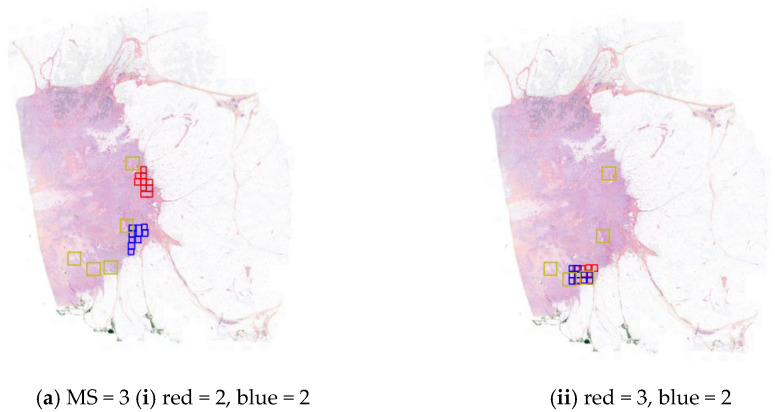

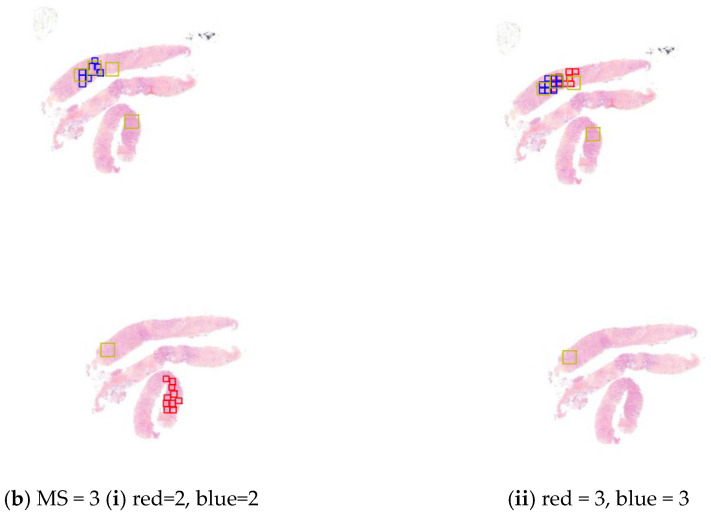
Observed changes in MS and counting zones for both readers with and without AI. Surgical specimen (**a**) and biopsy (**b**) with consensus expert MS = 3. (**i**) (readers without AI assistance) and (**ii**) (readers assisted by AI); AI hotspots in yellow, and counting zones and MS obtained defined by both readers in blue and red.

The agreement between the investigators’ chosen counting zones and the AI-suggested hotspots also increased significantly. The proportion of slides where an investigator’s zone intersected an AI hotspot zone increased from 46% to 80% for investigator 1 and from 62% to 90% for investigator 2. Figure 4 illustrates various intersection scenarios observed in both biopsies and surgical WSIs, including AI assistance in locating mitotic hotspots (Figure 4a) and refining mitotic counts even in cases where significant hotspots were identified (Figure 4b).

## 5. Discussion

This clinical study demonstrates that our AI-based mitosis detection pipeline is an effective support tool for pathologists assessing MS. With AI assistance, pathologists achieved both improved accuracy and reproducibility.

Although the algorithm showed only moderate recall and low precision (likely influenced by annotation noise in the training data), its level of agreement with experts was comparable to the agreement observed between pathologists themselves [39]. This suggests that traditional metrics like precision and recall, while informative, may not fully capture an algorithm’s clinical utility due to their sensitivity to subjective annotation biases. Moreover, stable results across two external datasets—MIDOG and MITOS-Atypia (metrics reported in Table 3)—confirm the algorithm’s generalizability, a key requirement for real-world deployment.

AI assistance notably improved MS accuracy, especially in diagnostically complex cases. For example, investigator 1’s accuracy increased from 62% without AI to 76% with assistance, and investigator 2’s from 64% to 78% (Figure 3a,b). These improvements exceed those reported by Pantanowitz et al. [27], where AI improved MS accuracy by 11.82% in small, predefined regions of interest. In contrast, our pipeline supports full-slide analysis, allowing pathologists to navigate the entire WSI without restriction, offering a more scalable and realistic approach for integration into clinical workflows.

Reproducibility, evaluated using Cohen’s kappa and ICC, also improved significantly with AI assistance. The agreement between investigators and consensus increased from low to moderate levels (kappa from 0.378 to 0.629 for investigator 1 and from 0.457 to 0.726 for investigator 2). ICC between investigators rose from 0.591 (CI: [0.375–0.746]) to 0.883 (CI: [0.803–0.932]), matching levels observed among experts (Figure 3c). These results align with Balkenhol et al. [29], who reported a +0.13 increase in MS agreement and a modest ICC gain for MC reproducibility with AI assistance.

Further analysis by MS subgroups revealed that AI support yielded the greatest improvements in more ambiguous cases. For slides with MS 2, accuracy increased fivefold with a corresponding kappa improvement of +0.31. For MS 3, accuracy more than doubled, with kappa increasing by +0.16 (Table 4). In contrast, MS 1 cases, which already showed high reproducibility, benefited less from AI assistance. This finding supports the idea that AI is particularly valuable for intermediate or high-grade cases (MS 2 or 3), where pathologist interpretations typically vary more, and identifying representative hotspots is harder.

Specimen type also played a role in diagnostic consistency. MS accuracy was generally higher in surgical specimens than biopsies, likely due to broader tissue context. However, AI assistance improved outcomes across both sample types: from 60% to 72% in biopsies and from 66% to 82% in surgical specimens (Table 5). Biopsies, being spatially constrained, led to more consistent hotspot selection, whereas surgical specimens showed greater benefit from AI-guided region localization due to the larger search area.

Importantly, while the AI proposed candidate hotspots, pathologists retained full autonomy in region selection. Nevertheless, the high rate at which investigators utilized AI-suggested hotspots (80% and 90% of cases for investigators 1 and 2, respectively) indicates the tool effectively guided their attention toward diagnostically relevant regions. The agreement between investigators on counting zones increased from 44% to 60% with AI assistance (Figure 3d), confirming that AI can reduce variability in hotspot selection. These results are in line with Balkenhol et al.’s findings [29], where strong inter-observer agreement (kappa = 0.814) was achieved, although that study was limited to standardized 2 mm^2^ regions—unlike the full-slide, unconstrained evaluation presented here.

In summary, this study provides strong evidence that AI-assisted mitosis detection can significantly enhance the accuracy and reproducibility of mitotic score assessments on breast cancer WSIs, particularly in diagnostically challenging cases. By evaluating the tool on whole slides in a realistic diagnostic setting, without pre-selecting regions, this work extends beyond prior studies focused on limited areas or more controlled environments. The crossover design and inclusion of both biopsies and surgical specimens offer a comprehensive view of the tool’s clinical applicability.

Compared to earlier research—such as that by Pantanowitz et al. [27] and Balkenhol et al. [29] (Table 6)—the present study distinguishes itself by evaluating AI assistance over the entire diagnostic workflow, allowing unrestricted region selection while still demonstrating substantial improvements in diagnostic performance. In doing so, it validates the algorithm’s utility in routine practice rather than under constrained experimental conditions. The results also highlight that even algorithms with moderate traditional detection metrics can provide meaningful clinical value, particularly when supported by evidence of expert-level agreement and generalizability across datasets.

Importantly, this work addresses a critical barrier to reproducible mitotic scoring—variability in region selection. By quantifying agreement on hotspot location and showing that AI assistance helps pathologists converge on similar areas without restricting their judgment, this study clarifies how AI can reduce inter-observer variability in both mitotic counts and overall scores.

Altogether, these findings support the analytical validity and clinical relevance of the proposed AI pipeline. They underscore its potential to serve as a reliable decision-support tool, particularly in cases with moderate to high mitotic activity, where pathologist agreement is most challenging. As such, this tool represents a promising avenue for standardizing mitotic scoring across institutions and enhancing diagnostic quality in breast cancer pathology.

## 6. Limitations and Further Works

This study has several limitations. First, despite our two-step expert annotation protocol, some errors (‘noise’) likely remain in the reference annotations. This is partly due to the inherent difficulty and known inter-observer variability in identifying mitoses, especially when comparing digital slides to glass slides [30,31,32,33,34]. Although consensus review using both modalities aimed to reduce false positives, some incorrect or missed annotations may persist, potentially impacting both the algorithm’s training and the accuracy of ground truth mitotic scores. To mitigate this bias, future efforts could involve a larger pool of expert pathologists to independently annotate and cross-verify mitotic figures. The incorporation of phosphohistone H3 (PHH3) immunohistochemistry as a supplementary reference standard could further enhance objectivity in mitotic figure identification, according to Corner et al.’s study [43]. Systematic comparison between digital and glass slide assessments may also help improve annotation reliability, particularly for borderline cases. Interestingly, a recent study on meningiomas by Haeri et al. [44] demonstrated that z-stack scanning—by capturing multiple focal planes—can significantly enhance AI mitosis detection sensitivity (+17.14%) across different scanner-AI setups. This suggests that such emerging technology could help bridge the performance gap between digital slides and conventional microscopy.

Second, the training dataset included a relatively limited number of annotated slides (50 from MIDOG and 32 from Bicêtre), which may constrain the diversity of morphological features, staining patterns, and artifacts encountered during algorithm training. While the inclusion of real-world slides from clinical practice aimed to increase variability, broader datasets incorporating multiple centers and scanners could further enhance generalizability. Additionally, data augmentation strategies, such as CycleGAN-based augmentation, could be employed to generate synthetic, diverse training samples, as seen in Nerrienet et al. [45]. This could help improve model performance and enhance the generalizability of the algorithm to a wider range of slide characteristics.

Third, the algorithm itself exhibited moderate recall and low precision, which may partly reflect the annotation biases in the training data. Furthermore, the algorithm’s false positives could create visual clutter for pathologists during review, potentially affecting usability or diagnostic confidence. Additional filtering strategies or interface refinements may help alleviate this issue.

Fourth, the clinical evaluation involved two investigators from a single institution. While the crossover design allowed robust intra-observer comparisons, generalizing these results to other institutions, workflows, or pathologists with different experience levels requires further confirmation. Including a broader panel of pathologists—both for algorithm validation and clinical evaluation—could help assess inter-observer robustness more comprehensively.

Finally, while the study design permitted unrestricted slide navigation to replicate real-world conditions, this freedom introduces variability in region selection. Although AI assistance improved consistency in hotspot identification, it remains challenging to fully isolate the effects of AI guidance from individual navigation strategies.

## 7. Conclusions

This study demonstrates that an AI-based mitosis detection pipeline can substantially improve both the accuracy and reproducibility of mitotic score assessment in breast cancer whole-slide images, particularly in diagnostically challenging cases. By guiding pathologists toward regions of high mitotic activity, the AI system improves consistency in hotspot selection and scoring without limiting the pathologists’ final judgment. These benefits were observed across different specimen types and mitotic score categories, with the most significant impact in intermediate and high mitotic activity cases—where interobserver variability is typically greatest.

Unlike prior studies focused on predefined regions, this work evaluates AI assistance on whole slides in a realistic setting, offering a more representative assessment of its clinical utility. The algorithm’s stable performance across diverse datasets and scanner types further supports its generalizability and potential for broader deployment.

However, this study also highlights challenges in validating digital pathology tools. Defining a reliable ground truth for subjective features like mitotic figures is difficult, as are potential discrepancies between digital and traditional microscopy assessments. Addressing these limitations will require comprehensive strategies such as involving multiple expert pathologists, comparing digital and glass slide interpretations, and incorporating objective references like PHH3 immunohistochemistry [43]. Moreover, recent advances in z-stack scanning [44], which improve sensitivity by capturing multiple focal planes, offer promising avenues to further align digital assessments with microscopic ground truth.

Altogether, these findings highlight the clinical relevance of explainable AI tools for mitotic scoring, especially in settings where diagnostic consistency is critical. By improving accuracy and reproducibility while preserving expert autonomy, the proposed pipeline represents a practical step toward a more standardized, AI-augmented pathology. Future work should aim to refine validation frameworks, extend evaluations across institutions, and integrate new imaging modalities to address the nuanced challenges of real-world diagnostic implementation.

## Figures and Tables

**Table 1:** Slides and patches distribution across datasets and tasks.

Slides	Training	12 (Bicêtre) and 150 (MIDOG21)			
	Testing	17 (Bicêtre)			
Patches		Detection		Classification	
	Size	256 × 256 pixel		50 × 50 pixel	
	Magnification	×20		×20	
	Class	Mitotic	Not mitotic	Mitosis	Artifacts
	number in training	2791	1341	3106	8638
	number in testing	146	24,716	153	5081

**Table 2:** Training settings and hyperparameters.

Model	Loss Function	Optimizer	Learning Rate Strategy	Hyperparameters
RetinaNet	L1 + Focal loss	SDG	Piecewise constant decay	LR = 0.01, momentum = 0.9, weight decay = 1 × 10^−4^
MobileNetV2	BCE	Adam	Constant	LR = 0.0001

**Table 3:** Detection metrics across datasets.

Dataset	Recall	Precision
Private	43.8%	27.6%
MIDOG 2022	33.1%	37.2%
MITOS-ATYPIA [19]	39.6%	28.6%

**Table 4:** Patient and tumor characteristics of the cohort used in the study.

Cohort (*n* = 50)	
	Number of Cases
Gender	
Female	50 (100%)
Male	0 (0%)
**Age**	
≥50 years	42 (84%)
<50 years	8 (16%)
**Pathological tumor stage** (for breast resection only—25 cases)	
pT1	18 (72%)
pT2	4 (16%)
pT3	1 (4%)
pT4	2 (8%)
**Pathological lymph node stage** (for breast resection only—25 cases)	
N0 (including isolated tumor cells)	12 (48%)
N1	9 (36%)
N2	0 (0%)
N3	1 (4%)
Nx	3 (12%)
**Histologic subtype**	
Invasive carcinoma of no special type	39 (78%)
* with neuroendocrine differentiation*	2 (4%)
Mixed invasive carcinoma of no special type	
* with mucinous carcinoma*	1 (2%)
* with invasive micropapillary carcinoma*	1 (2%)
Invasive lobular carcinoma	6 (12%)
Pure invasive micropapillary carcinoma	1 (2%)
**Tumor ER/PR and HER2 status**	
ER+/PR+/HER2-	39 (78%)
ER+/PR-/HER2-	6 (12%)
ER-/PR-/HER2-	2 (4%)
ER-/PR-/HER2+	3 (6%)
**Lymphovascular invasion**	
Negative	47 (94%)
Positive	3 (6%)
**In situ carcinoma associated**	
Yes	18 (36%)
No	32 (64%)
**Mitotic score**	
1	29 (55%)
2	12 (24%)
3	9 (18%)

Bold refers to characteristic name; *italic* refers to characteristic value.

**Table 5:** Metrics obtained at the subgroup level. Subgroups are defined by slide mitotic score and slide types—biopsies or specimens.

	Score 1 (n = 29)		Score 2 (n = 9)		Score 3 (n = 12)		Biopsies (n = 25)		Specimens (n = 25)	
AI assistance	No	Yes	No	Yes	No	Yes	No	Yes	No	Yes
Score accuracy (%)	94.83	94.83	5.56	33.33	29.17	66.67	60.00	72.00	66.00	82.00
Linear weighted CK	0.47	/	0	0.31	031	0.47	0.17	0.53	0.55	0.73
% of slides where readers’ counting zone intersect	48.3	48.3	44.4	55.6	33.3	66.7	32.0	60.0	56.0	60.0
% of slides where AI hotspot intersect										
Reader1’s counting zone Reader2’s counting zone	37.9 58.6	79.3 89.7	66.7 77.8	77.8 88.9	50.0 68.7	83.3 91.7	40.0 60.0	84.0 100.0	48.0 68.0	76.0 88.0

**Table 6:** Comparative table highlighting the similarities and differences between our findings and those of other studies.

Paper	Study Design	Automatic ROI Selection	Main Results
Balkenhol et al. (2019), [29]	Pathologists assessed semi automatically pre-extracted high-power fields (HPFs) MC with microscope vs. digital slides with AI assistance.	Partial	They demonstrate a +0.13 improvement in Cohen’s Kappa for MS agreement, and a +0.02 increase in ICC for MC agreement
Pantanowitz et al. (2020), [27]	Pathologists assessed pre-extracted high-power fields (HPFs) MS with and without AI assistance.	No	AI assistance led to an 11.82% increase in MS accuracy. The study focused on accuracy improvement when using AI in selected high-power fields.
van Bergeijk et al. (2023), [28]	Slide reading in a clinical setup, comparing microscope-based reading vs. WSI with and without AI assistance. Pathologists assessed mitotic count with and without AI.	Yes	AI-assisted mitotic count was found to be possibly non-inferior to conventional microscopic evaluation. The study suggests AI assistance could be integrated into clinical workflows.
Ours	Clinical study evaluating AI-assisted mitotic counting in a real-world setup. Pathologists analyzed WSI with and without AI assistance, and results were compared against ground truth. AI automatically selected hotspots.	Yes	Our study demonstrated a +14% increase in MS accuracy, a +0.19 improvement in Cohen’s Kappa for MS agreement, a +0.29 increase in ICC for MC agreement, and a +16% improvement in hotspot agreement, highlighting the benefits of AI assistance in mitotic counting.

**Figure A1:**
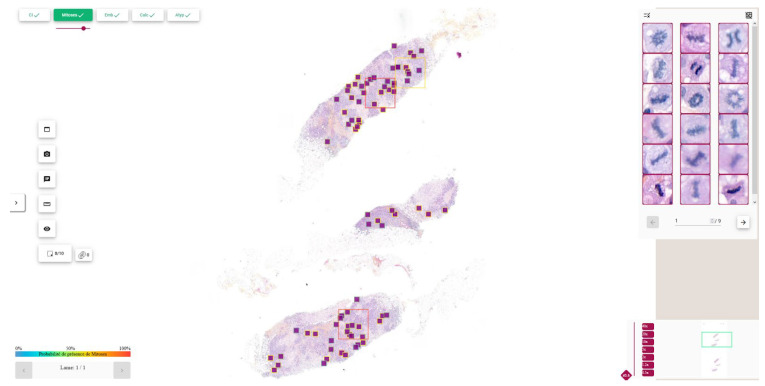

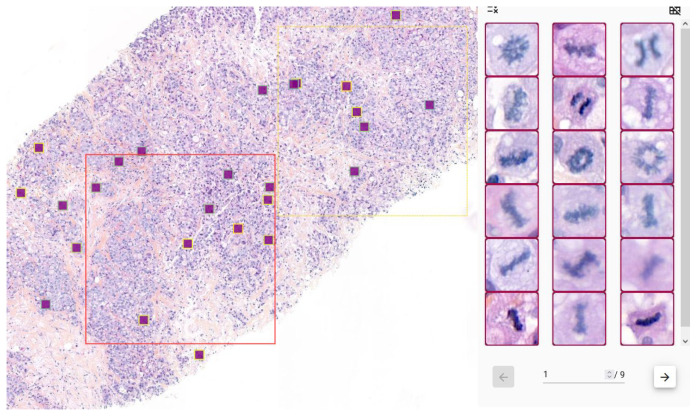
Results displayed in the in-house interface. Mitosis appear as small purple squares with contour colored based on their classification confidence score. They can also be seen in the right panel for easy identification navigation between objects. Hotspots are displayed as colored rectangles, with colors corresponding to their density score which lies between 0 and 100 (red is higher than yellow).

## Data Availability

The data presented in this study are available on request from the corresponding author.

## Abbreviations

The following abbreviations are used in this manuscript:

WSI	Whole Slide Images
IC-NST	Invasive Carcinoma of No Special Type
MS	Mitotic Score
MC	Mitotic Count
MH	Mitotic Hotspot
SFP	French Society of Pathology
CNN	Convolutional Neural Networks
HE	Hematoxylin Eosin
HES	Hematoxylin Eosin Safran
CI	Confidence Interval
ICC	Intraclass Correlation Coefficient
CK	Cohen’s Kappa

## Appendix A. Analytical Validation of the Mitosis Detection Algorithm

### Appendix A.1. Objective

In order to evaluate the performance of a mitosis detection algorithm on histological slides of invasive breast carcinoma, four pathologists independently assessed 50 slides of invasive ductal carcinoma of the breast. Each pathologist reviewed predefined 2 mm^2^ tumor through a dedicated user interface displaying detected mitoses.

The evaluation focused on the following:

- The number of mitoses counted by each pathologist.
- The mitotic score assigned (based on standard cut-offs).
- The agreement between mitoses annotated by pathologists and those detected by the algorithm.

### Appendix A.2. Key Results

***Pathologists and AI reproducibility:***

**Table A1:** Intraclass coefficient (ICC) and linearly weighted Cohen’s kappa (CK) coefficients for the evaluation of the mitotic score between each pathologist and the algorithm and between all pathologists.

		Pathologist 1	Pathologist 2	Pathologist 3	Pathologist 4
Algorithm	ICC	0.45 [0.10–0.68]	0.67 [0.49–0.80]	0.68 [0.50–0.80]	0.77 [0.627–0.86]
	CK	0.42	0.58	0.62	0.69
Pathologist1	ICC		0.48 [0.12–0.70]	0.78 [0.15–0.92]	0.70 [0.17–0.88]
	CK		0.34	0.47	0.47
Pathologist2	ICC			0.77 [0.63–0.87]	0.80 [0.67–0.89]
	CK			0.66	0.66
Pathologist3	ICC				0.90 [0.83–0.94]
	CK				0.75
Pathologist4	ICC				
	CK				

***Consensus on mitotic annotations*:** 52% of mitoses were annotated by at least two pathologists, while only 14% were marked by all four observers. ***Algorithm performance*:** The algorithm showed a mitosis recognition capability comparable to that of the pathologists. For three out of four observers, mitotic counts and corresponding scores were similar to those predicted by the algorithm.

### Appendix A.3. Conclusions

The algorithm demonstrated a performance level comparable to that of human experts in detecting mitotic figures.

## Appendix B. Raw Confusion Matrices

**Table A2:** Cross-comparisons for inter-observer agreement for mitotic scores between investigator 1 and expert consensus with and without the algorithm.

**Expert consensus**	**Pathologist 1 without algorithm**				
	Score	1	2	3	Total
	1	27	2	0	29
	2	7	1	1	9
	3	4	5	3	12
	Total	38	8	4	50
Linearly weighted Cohen’s Kappa was 0.378 (95% CI 0.179–0.577) without the use of the algorithm.					
**Expert consensus**	**Pathologist 1 with algorithm**				
	Score	**1**	**2**	**3**	Total
	**1**	27	1	1	29
	**2**	4	4	1	9
	**3**	2	3	7	12
	Total	33	8	9	50
Linearly weighted Cohen’s Kappa was 0.629 (95% CI 0.437–0.820) with the use of the algorithm.					

**Table A3:** Cross-comparisons for inter-observer agreement for mitotic scores between investigator 2 and expert consensus with and without the algorithm.

**Expert consensus**	**Pathologist 2 without algorithm**				
	Score	**1**	**2**	**3**	Total
	**1**	28	1	0	29
	**2**	9	0	0	9
	**3**	2	6	4	12
	Total	39	7	4	50
Linearly weighted Cohen’s Kappa was 0.457 (95% CI 0.267–0.647) without the use of the algorithm.					
**Expert consensus**	**Pathologist 2 with algorithm**				
	Score	**1**	**2**	**3**	Total
	**1**	28	1	0	29
	**2**	7	2	0	9
	**3**	0	3	9	12
	Total	35	6	9	50
Linearly weighted Cohen’s Kappa was 0.726 (95% CI 0.575–0.876) with the use of the algorithm.					

**Table A4:** Cross-comparisons for inter-observer agreement for mitotic scores between investigators 1 and 2 without the algorithm.

**Pathologist 2 Without Algorithm**	**Pathologist 1 without algorithm**				
	Score	**1**	**2**	**3**	Total
	**1**	34	4	1	39
	**2**	4	2	1	7
	**3**	0	2	2	4
	Total	38	8	4	50
Linearly weighted Cohen’s Kappa was 0.482 (95% CI 0.231–0.733) without the use of the algorithm. Intraclass correlation coefficient for mitotic count without algorithm: 0.591 (95% CI 0.375–0.746)					

**Table A5:** Cross-comparisons for inter-observer agreement for mitotic scores between investigators 1 and 2 with the algorithm.

**Pathologist 2 with Algorithm**	**Pathologist 1 with algorithm**				
	Score	**1**	**2**	**3**	Total
	**1**	30	4	1	35
	**2**	1	4	1	6
	**3**	2	0	7	9
	Total	33	8	9	50
Linearly weighted Cohen’s Kappa was 0.672 (95% CI 0.461–0.882) with the use of the algorithm. Intraclass correlation coefficient for mitotic count without algorithm: 0.883 (95% CI 0.803–0.932)					

## References

[1] Garcia E., Kundu I., Kelly M., Soles R., Mulder L., Talmon G.A. (2020). The American Society for Clinical Pathology’s Job Satisfaction, Well-Being, and Burnout Survey of Pathologists. Am. J. Clin. Pathol..

[2] Cruz-Roa A., Basavanhally A., González F., Gilmore H., Feldman M., Ganesan S., Shih N., Tomaszewski J., Madabhushi A., Gurcan M.N., Madabhushi A. (2014). Automatic Detection of Invasive Ductal Carcinoma in Whole Slide Images with Convolutional Neural Networks. Proceedings of the SPIE Proceedings.

[3] Celik Y., Talo M., Yildirim O., Karabatak M., Acharya U.R. (2020). Automated Invasive Ductal Carcinoma Detection Based Using Deep Transfer Learning with Whole-Slide Images. Pattern Recognit. Lett..

[4] Peyret R., Pozin N., Sockeel S., Kammerer-Jacquet S.-F., Adam J., Bocciarelli C., Ditchi Y., Bontoux C., Depoilly T., Guichard L. (2023). Multicenter Automatic Detection of Invasive Carcinoma on Breast Whole Slide Images. PLoS Digit. Health.

[5] Sun P., He J., Chao X., Chen K., Xu Y., Huang Q., Yun J., Li M., Luo R., Kuang J. (2021). A Computational Tumor-Infiltrating Lymphocyte Assessment Method Comparable with Visual Reporting Guidelines for Triple-Negative Breast Cancer. EBioMedicine.

[6] Elston C.W., Ellis I.O. (2002). Pathological Prognostic Factors in Breast Cancer. I. The Value of Histological Grade in Breast Cancer: Experience from a Large Study with Long-Term Follow-up. Histopathology.

[7] Rakha E.A., Bennett R., Coleman D., Pinder S.E., Ellis I.O. (2016). Review of the National External Quality Assessment (EQA) Scheme for Breast Pathology in the UK. J. Clin. Pathol..

[8] Berbís M.A., McClintock D.S., Bychkov A., Van Der Laak J., Pantanowitz L., Lennerz J.K., Cheng J.Y., Delahunt B., Egevad L., Eloy C. (2023). Computational Pathology in 2030: A Delphi Study Forecasting the Role of AI in Pathology within the next Decade. eBioMedicine.

[9] Irshad H. (2013). Automated Mitosis Detection in Histopathology Using Morphological and Multi-Channel Statistics Features. J. Pathol. Inform..

[10] Tek F.B. (2013). Mitosis Detection Using Generic Features and an Ensemble of Cascade Adaboosts. J. Pathol. Inform..

[11] Mathew T., Kini J.R., Rajan J. (2021). Computational Methods for Automated Mitosis Detection in Histopathology Images: A Review. Biocybern. Biomed. Eng..

[12] Ibrahim A., Lashen A., Katayama A., Mihai R., Ball G., Toss M., Rakha E. (2021). Defining the Area of Mitoses Counting in Invasive Breast Cancer Using Whole Slide Image. Mod. Pathol..

[13] Razavi S., Dambandkhameneh F., Androutsos D., Done S., Khademi A. (2021). Cascade RCNN for MIDOG Challenge. International Conference on Medical Image Computing and Computer-Assisted Intervention.

[14] Wilm F., Marzahl C., Breininger K., Aubreville M. (2021). Domain Adversarial RetinaNet as a Reference Algorithm for the MItosis DOmain Generalization Challenge. International Conference on Medical Image Computing and Computer-Assisted Intervention.

[15] Yang S., Luo F., Zhang J., Wang X. (2021). Sk-Unet Model with Fourier Domain for Mitosis Detection. International Conference on Medical Image Computing and Computer-Assisted Intervention.

[16] Roy G., Dedieu J., Bertrand C., Moshayedi A., Mammadov A., Petit S., Hadj S.B., Fick R.H.J. (2021). Robust Mitosis Detection Using a Cascade Mask-RCNN Approach With Domain-Specific Residual Cycle-GAN Data Augmentation. arXiv.

[17] Kausar T., Wang M., Ashraf M.A., Kausar A. (2021). SmallMitosis: Small Size Mitotic Cells Detection in Breast Histopathology Images. IEEE Access.

[18] Sebai M., Wang X., Wang T. (2020). MaskMitosis: A Deep Learning Framework for Fully Supervised, Weakly Supervised, and Unsupervised Mitosis Detection in Histopathology Images. Med. Biol. Eng. Comput..

[19] Aubreville M., Bertram C., Marzahl C., Maier A., Klopfleisch R. (2019). A Large-Scale Dataset for Mitotic Figure Assessment on Whole Slide Images of Canine Cutaneous Mast Cell Tumor. Sci. Data.

[20] Mitosis Detection in Breast Cancer Histological Images (MITOS Dataset). http://ludo17.free.fr/mitos_2012/dataset.html.

[21] Mitos Atypia 14 Contest. https://mitos-atypia-14.grand-challenge.org/.

[22] Aubreville M. (2023). Mitosis Domain Generalization in Histopathology Images—The MIDOG Challenge. Med. Image Anal..

[23] Li Z., Li X., Wu W., Lyu H., Tang X., Zhou C., Xu F., Luo B., Jiang Y., Liu X. (2024). A Novel Dilated Contextual Attention Module for Breast Cancer Mitosis Cell Detection. Front. Physiol..

[24] Subramanian R., Rubi R.D., Tapadia R., Karthik K., Ahmed M.F., Manudeep A. (2022). Web Based Mitosis Detection on Breast Cancer Whole Slide Images Using Faster R-CNN and YOLOv5. Int. J. Adv. Comput. Sci. Appl..

[25] Jahanifar M., Shephard A., Zamanitajeddin N., Graham S., Raza S.E.A., Minhas F., Rajpoot N. (2024). Mitosis Detection, Fast and Slow: Robust and Efficient Detection of Mitotic Figures. Med. Image Anal..

[26] Wang H., Liu Z., Pan X., Yu K., Lan R., Guan J., Li B. (2025). A Novel Dataset and a Two-Stage Deep Learning Method for Breast Cancer Mitosis Nuclei Identification. Digit. Signal Process..

[27] Pantanowitz L.X., Hartman D.J., Qi Y., Cho E.Y., Suh B., Paeng K., Dhir R., Michelow P.M., Hazelhurst S., Song S.Y. (2020). Accuracy and Efficiency of an Artificial Intelligence Tool When Counting Breast Mitoses. Diagn. Pathol..

[28] van Bergeijk S.A., Stathonikos N., Hoeve N.D., Lafarge M.W., Nguyen T.Q., van Diest P.J., Veta M. (2023). Deep Learning Supported Mitoses Counting on Whole Slide Images: A Pilot Study for Validating Breast Cancer Grading in the Clinical Workflow. J. Pathol. Inform..

[29] Balkenhol M.C.A., Tellez D., Vreuls W., Clahsen P.C., Pinckaers H., Ciompi F., Bult P., van der Laak J.A.W.M. (2019). Deep Learning Assisted Mitotic Counting for Breast Cancer. Lab. Investig..

[30] Ibrahim A., Lashen A., Toss M., Mihai R., Rakha E. (2022). Assessment of Mitotic Activity in Breast Cancer: Revisited in the Digital Pathology Era. J. Clin. Pathol..

[31] Williams B., Hanby A., Millican-Slater R., Verghese E., Nijhawan A., Wilson I., Besusparis J., Clark D., Snead D., Rakha E. (2020). Digital Pathology for Primary Diagnosis of Screen-Detected Breast Lesions—Experimental Data, Validation and Experience from Four Centres. Histopathology.

[32] Shaker O.G., Kamel L.H., Morad M.A., Shalaby S.M. (2020). Reproducibility of Mitosis Counting in Breast Cancer between Whole Slide Images and Glass Slides. Pathol. Res. Pract..

[33] Ginter P.S., Lee Y.J., Suresh A., Acs G., Yan S., Reisenbichler E.S. (2021). Mitotic Count Assessment on Whole Slide Images of Breast Cancer: A Comparative Study with Conventional Light Microscopy. Am. J. Surg. Pathol..

[34] Rakha E.A., Toss M.S., Al-Khawaja D., Mudaliar K., Gosney J.R., Ellis I.O., Dalton L.W. (2018). Impact of Whole Slide Imaging on Mitotic Count and Grading of Breast Cancer: A Multi-Institutional Concordance Study. J. Clin. Pathol..

[35] Cytomine. https://cytomine.com/.

[36] Lin T.-Y., Goyal P., Girshick R., He K., Dollár P. Focal Loss for Dense Object Detection. Proceedings of the IEEE International Conference on Computer Vision.

[37] Howard A.G., Zhu M., Chen B., Kalenichenko D., Wang W., Weyand T., Andreetto M., Adam H. (2017). MobileNets: Efficient Convolutional Neural Networks for Mobile Vision Applications. arXiv.

[38] Kingma D.P., Ba J. (2014). Adam: A Method for Stochastic Optimization. arXiv.

[39] Guichard L. (2022). Evaluation du Score Mitotique des Carcinomes Mammaires Infiltrants: Développement et Apport d’un Algorithme de Détection de Mitoses. PhD Thesis.

[40] Aubreville M., Stathonikos N., Donovan T.A., Klopfleisch R., Ammeling J., Ganz J., Wilm F., Veta M., Jabari S., Eckstein M. (2024). Domain Generalization across Tumor Types, Laboratories, and Species—Insights from the 2022 Edition of the Mitosis Domain Generalization Challenge. Med. Image Anal..

[41] Koo T.K., Li M.Y. (2016). A Guideline of Selecting and Reporting Intraclass Correlation Coefficients for Reliability Research. J. Chiropr. Med..

[42] McHugh M.L. (2012). Interrater Reliability: The Kappa Statistic. Biochem. Medica.

[43] Bertram C.A., Aubreville M., Donovan T.A., Bartel A., Wilm F., Marzahl C., Assenmacher C.-A., Becker K., Bennett M., Corner S. (2022). Computer-Assisted Mitotic Count Using a Deep Learning–Based Algorithm Improves Interobserver Reproducibility and Accuracy. Vet. Pathol..

[44] Gu H., Onstott E., Yan W., Xu T., Wang R., Wu Z., Chen X.A., Haeri M. (2025). Z-Stack Scanning Can Improve AI Detection of Mitosis: A Case Study of Meningiomas. arXiv.

[45] Nerrienet N., Peyret R., Sockeel M., Sockeel S. (2023). Standardized CycleGAN Training for Unsupervised Stain Adaptation in Invasive Carcinoma Classification for Breast Histopathology. J. Med. Imaging.

